# Intermolecular dark resonance energy transfer (DRET) for high contrast imaging of endogenous mRNAs in 3D biological samples

**DOI:** 10.1038/s41598-025-27000-1

**Published:** 2025-12-01

**Authors:** Guillaume Barnoin, Fabienne De Graeve, Aurélien Darnas, Sameh Ben Aicha, Baptiste Monterroso, Alain Burger, Florence Besse, Benoît Y. Michel

**Affiliations:** 1 Université Côte d’Azur, CNRS, Institut de Chimie de Nice, Nice, France; 2 Université Côte d’Azur, CNRS, INSERM, Institut de Biologie Valrose (iBV), Nice, France

**Keywords:** Fluorogenic probes, RNA imaging, *Oskar* mRNA, DRET vs FRET, In situ hybridization, Biological techniques, Biophysics, Biotechnology

## Abstract

In cellulo detection of RNA molecules is a cornerstone for understanding many biological processes, with recent advances heavily centered on fluorescence-based strategies. The fluorescence resonance energy transfer process stands out as an indispensable tool due to the high specificity of the signal emitted by the acceptor when in proximity to the donor. However, inherent crosstalk between the spectral bands of FRET-compatible fluorophores often diminishes detection sensitivity. Capitalizing on the unique capacity of poorly emissive fluorophores to transfer their energy by resonance, we previously introduced the intermolecular Dark-RET (DRET) concept. This approach has the advantage of minimizing the background noise while maximizing the fluorogenic response via the acceptor emission channel. In this study, we refined both theoretical and hands-on aspects of the DRET approach, providing a readily accessible framework to the scientific community. We further underscored its potential to generate a specific fluorogenic response upon target recognition by detecting the *oskar* mRNA in the biological context of *Drosophila* oocytes. Combining in situ hybridization experiments with confocal imaging, we demonstrated the ability of the intermolecular DRET approach to generate high signal-to-noise ratios and specific target recognition, highlighting the benefits of the intermolecular DRET strategy compared to standard FRET approaches.

**Supplementary Information:**

The online version contains supplementary material available at 10.1038/s41598-025-27000-1.

## Introduction

Recent discoveries highlighting the tight spatio-temporal regulation of subcellular RNA distribution across a multitude of biological processes have underscored the need to accurately detect cellular RNAs^1,2^. To achieve this, it is essential to selectively illuminate the RNA molecules of interest, thereby producing a signal distinct from the surrounding background noise^3^. Fluorescence-based strategies have been developed to visualize RNAs in cells^4^. Some of these approaches rely on the insertion of unique RNA tags recognized by specific trans-acting fluorophores^5–9^. Despite recent advances, such strategies face inherent limitations, including generation of high background signals from non-specific binding and potential alterations of target RNA natural behavior and interactions^1,9,10^.

Alternatively, RNAs can be directly detected in cells without genomic manipulation using antisense oligonucleotide probes that are capable of specific binding through Watson–Crick base pairing. The pioneering technique in this field is Fluorescence in situ Hybridization (FISH). Commonly used FISH fluorophores are highly bright and generally exhibit similar fluorescence properties on and off target. Therefore, increasing the number of labeled probes per RNA target is essential in order to create a bright focal spot that stands out against the background noise. This enables the accumulation of sufficient signal to detect an RNA sequence at the single-molecule level (smFISH)^11–13^.

Enhanced signal-to-noise ratio (SNR) can be attained for detection of cellular RNAs with fluorogenic oligonucleotide probes, which increase their fluorescence upon hybridization. By decreasing the number of required probes, this technique not only reduces cost, but also enables the imaging of a broad spectrum of RNA types, including small RNAs such as miRNA and siRNA, that are critical in gene regulation and numerous pathologies. A pivotal photophysical process employed in this context is Fluorescence Resonance Energy Transfer (FRET). This approach relies on the synergistic operation of two probes—a donor and an acceptor—to yield a specific signal on the target. In this process, target recognition is achieved either by the red-shifted acceptor emission triggered by the energy transfer from the donor, or by the ratio of their fluorescence emission intensities^14–16^. However, the sensitivity of FRET detection is hindered by the crosstalk between the absorption and emission bands of the two partners^17–19^. For instance, when the absorption spectra of the molecules overlap, the excitation of the donor directly triggers the acceptor (*cross-excitation*), leading to increased off-target signals and thereby to a decrease in SNR. Likewise, when their emission spectra overlap (*cross-emission*), the complete recovery of the acceptor’s fluorescence signal is impeded. This requires the use of highly selective filters to collect a spectral window uncontaminated by the fluorescence of the donor.

This limitation applies primarily to intensity-based FRET approaches and can be circumvented by measuring changes in donor lifetime using fluorescence lifetime imaging (FLIM-FRET), which reduces the dependency on stringent filter selection. However, this method entails additional hardware requirements and advanced decay-fitting analyses^20^. Indeed, since the photophysical response of fluorescent probes is strongly influenced by their local environment, lifetime measurements often display multi-exponential decays that are challenging to interpret unambiguously.

In the pursuit of enhanced performance, another strategy has involved the development of molecular beacons (MBs)—secondary nucleic acid structures in a stem-loop conformation that link a fluorophore and its quencher. Prior to hybridization with the target, the MB is in a closed state, silencing the fluorescence signal^21^. Upon annealing, the hairpin unfolds, restoring the donor’s emission. However, regulating the specific unfolding of the beacon remains challenging, as it can result either to false positives due to uncontrolled opening, or to a lack of signal if the stem-loop stays closed^22,23^.

**Fig. 1:**
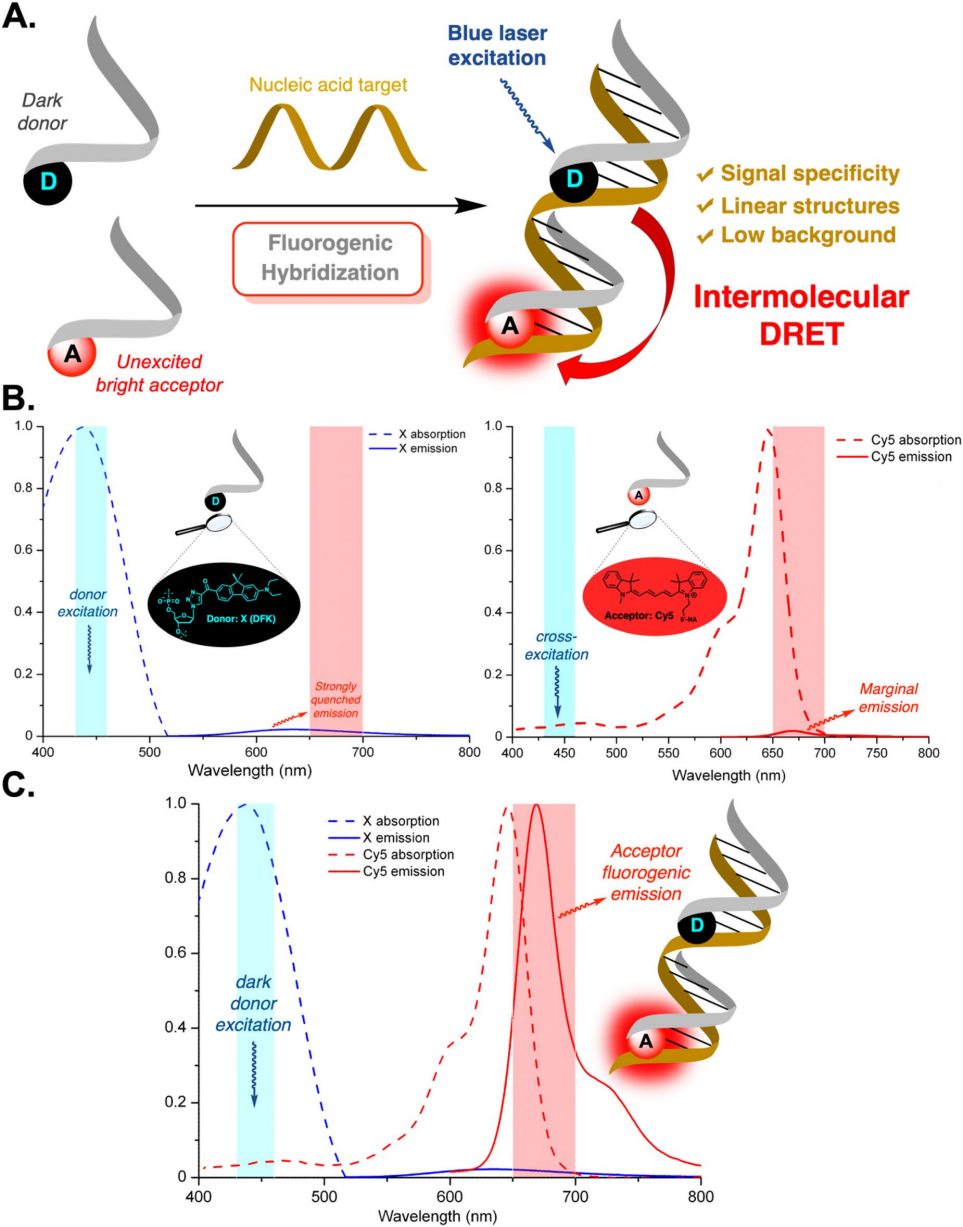
Intermolecular DRET strategy: concept, probe properties and spectral design. (**A**) Schematic representation of the reported intermolecular DRET for nucleic acid detection and its intrinsic properties. (**B**) Off-target photophysics of the **X**-donor (*left*) and **Cy5**-acceptor (*right*) probes. (**C**) Spectroscopic adaptation of the DRET fluorogenic concept based on the absorption and emission spectra of the developed **X**/**Cy5** DRET pair. *Highly selective blue-laser excitation of X induces the turn-on emission of Cy5 in the far-red.*

Thus, the detection and localization of RNAs within cells remains a field of research where significant advances are still awaited. To this end, we have recently developed a novel binary probe approach based on the dark-resonance energy transfer (DRET) process (Fig. 1A)^24^. The ability to perform Resonance Energy Transfer from a quenched donor was first evidenced by Lakowicz and coll.^25^. This concept has been used in an intramolecular version for the design of efficient fluorophores in small-molecule sensing, multiplexing or for probing the polarity and pH of a solution^26–30^. By adapting DRET to intermolecular sensing, we have successfully demonstrated its strong potential for both DNA and RNA detection in vitro. Compared to FRET, DRET almost annihilates the fluorescent signal of the donor and thus prevents cross-emission. Additionally, cross-excitation can be managed by using a quenched donor with a mega-Stokes shift (*Δ*λ > 100 nm). Since these photophysical properties are typically those expressed by push–pull dyes in water^31–34^, a screening of fluorenes with a D-*π*-A structure led to the design of the push–pull fluorene **DFK** (*ε* = 42,000 M^−1^·cm^−1^ in acetonitrile), a modified nucleoside with a very low quantum yield in water (< 1%) and a tremendous Stokes shift (> 240 nm) (Supplementary Fig. S1)^35–37^.

The principle of NA detection via intermolecular DRET mirrors that of linear binary FRET probes but has the substantial advantage of entirely suppressing the signal prior to annealing of the two probes. This is because the donor remains dark and the acceptor unexcited. Upon hybridization, the donor can transmit its energy through resonance to the acceptor, thereby illuminating the target. This strategy substantially diminishes the basal signal without requiring a quencher embedded within a complex structure, while maintaining the specificity and simplicity inherent to linear binary probes. Given that the acceptor’s fluorescent signal originates solely from the donor’s energy transfer, false positive signals are efficiently eliminated. Although designed for a similar purpose, donor turn-off FRET approaches that use a dark acceptor to alleviate spectral crowding are vulnerable to non-RET–based reductions in donor brightness (e.g., photobleaching, triplet/dark-state kinetics, variability in illumination or expression), which can mimic a FRET signature and inflate false-positive rates^38^.

By associating the **DFK** donor (named **X** once labeled) with the **Cy5** acceptor in cuvettes, we showed that it was possible to reach fluorescence amplification factors greater than 100 in the far-red and that—through the excitation of a quenched donor—the intrinsic fluorescence potential of the acceptor could be fully recovered. With its exquisite molar absorption coefficient (250,000 M^-1^·cm^-1^) as well as its weak fluorescence emission across a wide range of excitation wavelengths (430–460 nm), the **Cy5** dye proved to be perfectly adapted to this approach (Fig. 1B–C)^24^. Our next endeavor was to demonstrate the efficiency of DRET in detecting NAs within biological samples. For this purpose, we chose the *oskar* (*osk*) mRNA located at the posterior pole of the oocyte within *Drosophila* ovarian follicles. This mRNA, spanning approximately 4000 nucleotides, has been extensively studied by various groups^39–43^. RNA secondary structure prediction programs and in vitro hybridization experiments identified accessible regions, making *osk* mRNA a well-suited model for investigation^42^.

Herein, we unravel the mechanistic bases of intermolecular DRET to facilitate the design of potent DRET pairs and widen the accessibility of this technology. We focus on the evaluation of binary 2′-MeO-RNA probes, incorporating the quenched **X** donor and the bright **Cy5** acceptor, through meticulous in vitro studies on a segment of *osk* mRNA. We then describe FISH experiments performed in *Drosophila* ovarian follicles to compare the performance of FRET and DRET by confocal microscopy. The conventional **Cy3/Cy5** FRET pair detected *osk* mRNA, but suffered from substantial background arising from emissive unhybridized probes. In contrast, intermolecular DRET efficiently and specifically detected RNA molecules at the oocyte posterior pole while producing a notably lower background interference.

## Results & discussion

### Concept of intermolecular DRET: specifics and prerequisites

This section aims at providing a theoretical framework for the energy transfer process involving weakly fluorescent species and emphasizes the optimal photophysical characteristics of the donor probe upon hybridization to the target.

**Fig. 2:**
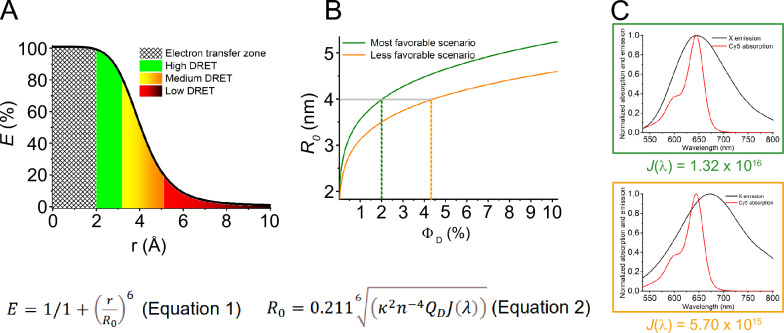
Theoretical and spectral parameters governing DRET efficiency. (**A**) Variation of the transfer efficiency as a function of the donor–acceptor distance considering a *R*_0_ value of 4 nm. *Förster’s theory states that a transfer efficiency close to 100% requires a donor–acceptor distance approximately less than or equal to half of the R*_*0*_. (**B**) Variation of *R*_0_ as a function of the donor quantum yield (*Φ*_D_ in the range 0–10%) for the **X**/**Cy5** pair comprising *κ* = 2/3, *n* = 1.4, and the corresponding-colored *J(λ)*. The green and orange lines represent the most favorable and less favorable cases with a **X**-emission maximum set at 644 and 674 nm, respectively. (**C**) Optical observables (X emission in black and Cy5 absorption in red) used for the determination of the overlap integrals (*J(λ)*) corresponding to the most favorable (green frame) and less favorable (orange frame) scenarios.

Successful sensing with the linear binary DRET probes approach requires a donor as quenched as possible in a single-stranded context. Upon hybridization, it should act as an antenna that can efficiently transfer energy to a bright acceptor. A donor in a complete fluorescence OFF state cannot fulfill this role, as its quantum efficiency (*Φ*_D_) is integral to the physical equations related to RET (Eqs in Fig. 2). In particular, this variable significantly influences the *R*_0_ calculation (*Förster radius*), which represents the donor–acceptor distance at which the transfer efficiency is 50%^44^. The fluorogenic response arising from intermolecular DRET is exclusively detected via the acceptor emission channel. Therefore, it is imperative that the energy transfer process approaches an efficiency of nearly 100%. The efficiency of the transfer is determined by the distance between the donor and acceptor, reaching close to 100% when the distance is less than or equal to *R*_0_/2 (Fig. 2A and Eq. 1). Given the potential interference from secondary photophysical processes like DEXTER (electron exchange energy transfer) or PET (photoinduced electron transfer), which can compete with RET at shorter distances, it is advised to maintain a donor–acceptor distance of more than 2 nm^45^. Optimal donor–acceptor pairs will thus have an R_0_ of at least 4 nm (Fig. 2A). Accordingly, a higher *R*_0_ facilitates the donor’s ability to transfer its energy at optimal efficiency over a distance where RET emerges as the fastest de-excitation pathway (Supplementary Fig. S1).

To fully exploit the potential of the intermolecular DRET approach, it is essential to achieve two distinct fluorescence states for the donor. Firstly, a **dark state in the unbound form** to eliminate background noise. Secondly, upon annealing to the complementary strand, the donor must become weakly emissive via a fluorogenic mechanism—just sufficient to push the Förster radius above 4 nm, thereby enabling efficient energy transfer to the acceptor.

Besides the donor’s quantum yield, the *R*_0_ value also depends on the integral overlap between the donor emission and the acceptor absorption, as well as the orientation of their transition dipoles. For the DRET **X**/**Cy5** pair, this spectral overlap is substantial and offsets the low quantum yield of the donor. Assuming a random orientation for the transition dipoles (*κ* = 2/3), a simulation of *R*_0_ variation in relation to the quantum yield (Φ_D_) was undertaken according to two spectral overlap *J*(*Φ*) scenarios (Fig. 2B,C and Eq. 2). In the most favorable scenario (represented by the green curve in panel B and the green box in panel C), the fluorescence emission of **X** coincides with the **Cy5** absorption peak (*λ*_Abs_ = 644 nm), maximizing *J*(*λ*). In the less favorable scenario (represented by the orange curve in panel B and the orange box in panel C), the donor exhibits a red-shifted emission (*λ*_Em_ = 674 nm). To ensure *R*_0_ exceeds 4 nm, the donor quantum yield *Φ*_D_ must range between 2.0–4.5%, depending on the magnitude of the spectral overlap integral *J*(*λ*) (as indicated by the dashed lines in Fig. 2B).

### In vitro analysis of *osk* RNA hybridization

In earlier research, the photophysical properties of the **X**-donor when incorporated into DNA probes revealed that this fluorophore is highly sensitive to its surrounding environment. Specifically, its quantum yield ranged between 0.7–2.2% in single strands and 1.2–6.7% in duplexes, depending on the nature of adjacent and opposite bases^24^. This fluctuation was found to be directly associated with the hydration level within the solvation sphere of the push–pull fluorene dye (Supplementary Fig. S2). A higher hydration rate corresponded to a lower donor quantum yield and red shifted emission. Consequently, to significantly minimize background noise, a highly hydrated milieu around this fluorophore in its single-stranded state is imperative. To achieve this, various base compositions were examined. The optimal outcome was obtained when the donor was positioned internally, flanked by multiple adenosines on both sides (*Φ* = 0.7%, Supplementary Fig. S3A)^24^. To maximize the fluorogenic effect upon hybridization, the most effective approach involved positioning a cytidine opposite to the **X-donor** in the complementary strand. This nucleobase is recognized for its propensity to flip out of the double helix, facilitating the proper intercalation of the donor into the duplex (*Φ* = 6.7%, Supplementary Fig. S3B). In this arrangement, the donor remains insulated from external water exposure. As a result, both its quantum yield and the *R*_0_ value raise, allowing it to transfer its energy nearly completely to the acceptor (Supplementary Fig. S4).

Employing OligoDeoxyriboNucleotide (ODN) probes for the detection of RNA sequences within a cell may be hampered by the fact that DNA/RNA hybrid duplexes can be degraded by endogenous nucleases^46^. To take this into account and generate probes usable both in vitro and in vivo, we thus designed DRET oligonucleotide probes that target the cellular *osk* mRNA with 2′-OMe-OligoRiboNucleotide (ORN) backbones^47^.

**Fig. 3:**
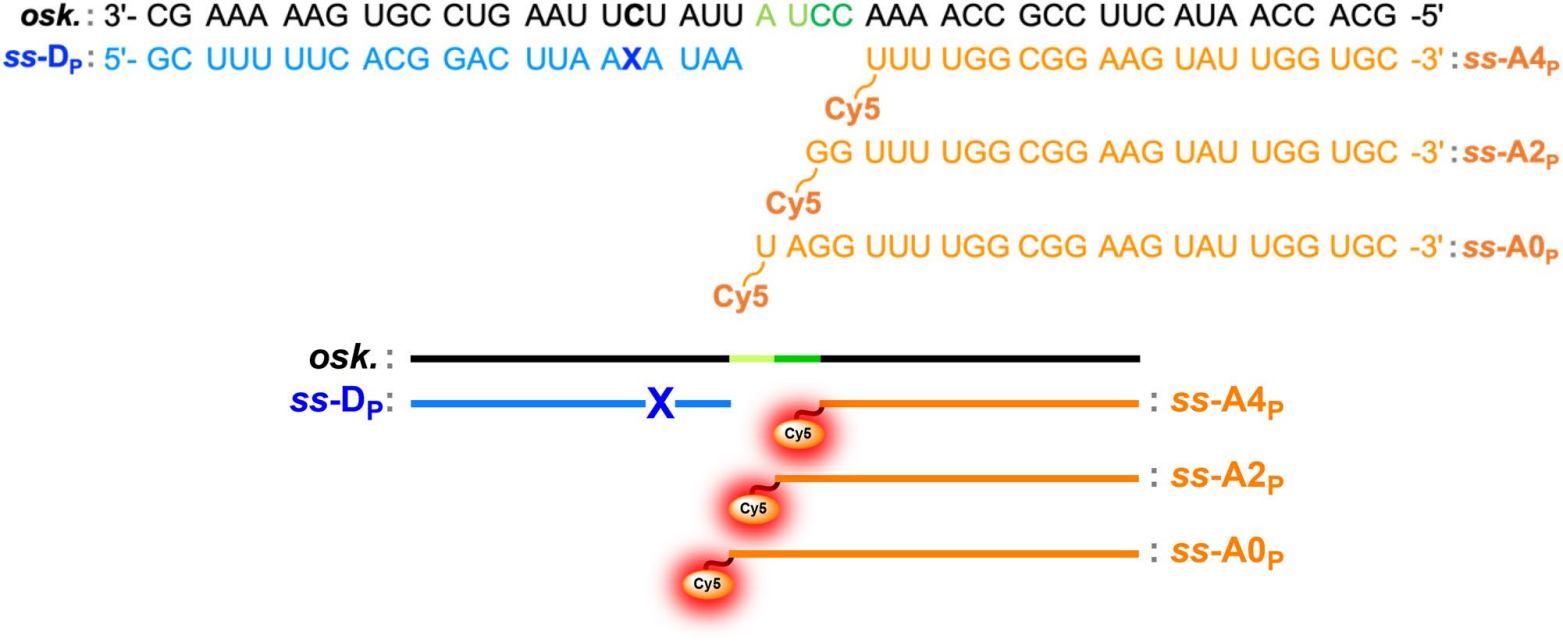
Design of linear binary DRET probes targeting *osk* mRNA. Representation of the Acceptor and Donor probes used to recognize the *osk* mRNA (in black on top). **A**_**p**_ and **D**_**P**_ correspond to acceptor and donor probes labeled with **Cy5** and **X**, respectively. They are complementary to the target sequence while assembling as either a nicked (**A0**) or a 2-/4-nucleotide gapped junction (**A2**/**A4**).

Taking advantage of previous studies^39,40,42^, an accessible segment of *osk* mRNA that meets the neighboring and opposite base criteria for effective **X** incorporation, was selected for preliminary in vitro investigations. Combining solid-phase synthesis with 2′-OMe-phosphoramidite chemistry, the **X**-modified amidite was inserted at the G position of an AGA antisense codon to prepare the corresponding donor probe (*ss*-**D**_**P **_, Fig. 3 and Supplementary Fig. S5). The labeled single strand was purified by RP-HPLC and characterized by UV–Vis spectroscopy, fluorescence, and MALDI-TOF mass spectrometry (Supplementary Figs. S4, S6 and Table S1).

The photophysical features of *ss*-**D**_**P**_ (*λ*_Abs_ = 435 nm; *λ*_Em_ = 655 nm) demonstrated that the **X**-fluorophore was subjected to a high-water exposure as attested by the low quantum yield of 1.6% and the mega-Stokes shift of 220 nm (Supplementary Figs. S7 and S8). Thermal denaturation studies further indicated that the ss-Dp/*osk* target duplex is well established at 25 °C (*T*_m_ = 62 °C, Supplementary Fig. S9). In addition, the donor photophysics was fully preserved upon hybridization (*λ*_Abs_ = 435 nm; *λ*_Em_ = 656 nm; Φ_D_ = 1.7%). In our previous work^24^, this fluorophore consistently exhibited a bathochromic shift and hyperchromism characteristic of effective donor intercalation upon DNA-DNA duplex formation. In contrast, absorption changes and altered fluorescence signature were not observed (Supplementary Fig. S7), which could be interpreted as a poor intercalation of the donor. As described below, the conformational changes in the duplex induced by modifications of the NA sugar backbone, must indeed be considered.

Compared with DNA/DNA duplexes, 2′-OMe-RNA/RNA duplexes adopt an A-form rather than a B-form geometry (See CD spectrum in Supplementary Fig. S10). The rise per base pair along the helical axis is reduced from 3.4 to 2.5 Å^48^, compacting the duplex and narrowing a less-hydrated major groove^49^. The close alignment of base pairs in this conformation restricts intercalation of the push–pull fluorene donor, leaving it water-exposed with a low quantum yield, which in turn reduces R₀ and, consequently, the maximum potential of RET efficiency. As described below, even under suboptimal conditions for intercalation, the DRET process remains efficient enough to produce strong fluorogenic amplification.

**Fig. 4:**
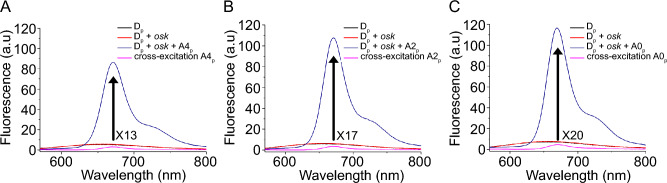
Fluorescence signal amplification in DRET ternary systems targeting *osk* mRNA. Fluorescence emission spectra of the single-stranded D_P_ in the absence of target RNA, the ds-construct in absence of acceptor (**D**_**P**_ + ***osk***) and the DRET-operating ternary systems: A. ([**D**_**P**_ + **A4**_**P**_] + ***osk***), B. ([**D**_**P**_ + **A2**_**P**_] + ***osk***), C. ([**D**_**P**_ + **A0**_**P**_] + ***osk***), and their corresponding acceptor cross-excitation. DRET between **X** and **Cy5** led to 13-, 17-, and 20-fold increases in the fluorescence sensing signal. Observables were recorded at 2 μM, in pH 7.4 PBS and excitation was performed at 458 nm corresponding to the blue laser wavelength used in the further imaging experiments. Fluorescence signal amplification was determined according to the following equation: S/N = (F_hybrid_—F_buffer_)/(F_ss_—F_buffer_) (Eq. 3)^63^.

In subsequent experiments, we evaluated how the distance between the donor and acceptor affects the DRET activation by hybridizing **Cy5** acceptor probes at different locations along the *osk sequence* (Fig. 3). When both partners were separated by a 4-nucleotide gap, a fluorescence amplification factor of 13 was observed (*determined by the intensity ratio at the Cy5 emission peak between the donor in a single-stranded context and the DRET ternary system*, Fig. 4A and Eq. 3). Reducing the gap to two nucleotides increased the amplification to 17 (Fig. 4B), and juxtaposing the strands via a nicked junction raised it to 20 (Fig. 4C). For this last combination, [**D**_**p**_** + A0**_**p**_], the DRET efficiency increased to 54% (Eq. S2 in Methods). This result is consistent with the theoretical Förster radius of 3.3 nm (calculated from Eq. 2 in Fig. 2).

To compare the performance of DRET and FRET probes, parallel studies were conducted using a 2′-OMe-ORN/ORN duplex labeled with the widely used **Cy3/Cy5** FRET pair. The chosen probes (**D**_**P-Cy3**_ and **A**_**P-Cy5**_, Supplementary Fig. S11) displayed a transfer efficiency of 55%, closely mirroring that of the previously studied DRET pair. In vitro analyses clearly showed the presence of *cross-talk*, a common FRET issue. This resulted in a modest amplification factor of 5 (Supplementary Fig. S11), primarily due to the residual **Cy3** donor fluorescence, highlighting the superior fluorogenic response of DRET probes.

### In situ* osk* mRNA detection and imaging

**Fig. 5:**
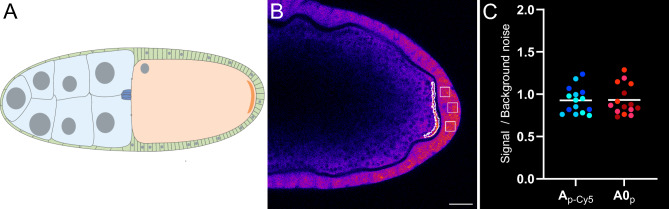
Organization of *Drosophila* egg chambers and signal quantification. (**A**) Schematic representation of a *Drosophila* stage-10A egg chamber, oriented from anterior (left) to posterior (right). The oocyte, shown in orange, is connected to the anteriorly localized nurse cells, depicted in blue. It is surrounded by follicular cells in green. Within the oocyte, *osk* mRNA predominantly accumulates at the posterior pole, forming a crescent (dark orange). (**B**) Single confocal section of a stage-10A egg chamber probed with the acceptor **A**_**P-Cy5**_. The areas measured to calculate the SNR are outlined in white. From left to right: the *osk* mRNA crescent (signal) and 3 square areas within the follicular cells (background noise). (**C**) Graph illustrating the distributions of signal-to-background intensity ratios after hybridization with Ap only. The mean grey values of the **Cy5** signal (ex. 633 nm, em. 650–700 nm) are derived from the FRET **A**_**P-Cy5**_ probes (left, shown in blue) and DRET **A0**_**P**_ (right, in red). These ratios were determined from 15 egg chambers across three independent experiments identified by specific shades of blue (left) or red (right). The white horizontal lines indicate average values. Scale bar shown in (B) represents 20 µm.

To assess the capacity of DRET probes to detect *osk* mRNA in biological samples, we performed in situ hybridizations on fixed and permeabilized *Drosophila* ovarian follicles (termed egg chambers), using the previously optimized DRET probes (**D**_**P**_ + **A0**_**P**_; Figs. 3 and 4C), as well as corresponding FRET probes (**D**_**P-Cy3**_ + **A**_**P-Cy5**_; Supplementary Fig. S11). As shown in Fig. 5A, each egg chamber is composed of one oocyte and its 15 sibling germ cells (the nurse cells) surrounded by somatic follicular cells^43^. *osk* mRNA is transcribed in the nurse cell and transported into the growing oocyte where it progressively accumulates. From stage 9 of development onwards, *osk* mRNA is mostly localized to the posterior pole of the oocyte, exhibiting an estimated local concentration above 300 nM. This transcript is found at a very low concentration in the remaining part of the oocyte (estimated around 0.8 nM) and is not expressed in follicular cells^42,50^.

In situ hybridizations were performed with a probe concentration of 0.3 μM and stage-10A egg chambers were imaged with a laser scanning confocal microscope. Fluorescence emission was collected between 650 and 700 nm. DRET analyses were conducted with an excitation at 458 nm, while FRET analyses were performed after excitation at 514 nm, owing to the low absorption coefficient of **Cy3** at 458 nm. Following the approach previously developed by Seitz et al.^39,40^, the average signal intensity of the oocyte posterior crescent was divided by that of the adjacent follicular cells to generate a signal:background ratio (or SBR) value used to compare samples (Fig. 5B). Acceptor probe distribution was assessed by directly exciting the **Cy5** acceptor with a red laser at 633 nm, close to **Cy5** absorption maximum at 644 nm. A background signal was discernible both at the posterior pole of the oocyte and within the follicular cells (Fig. 5B). Notably, for both acceptor probes (FRET **A**_**P-Cy5**_ & DRET **A0**_**P**_), the signal intensity was comparable at the oocyte posterior pole and in adjacent follicular cells, leading to a mean SNR close to 1 (Fig. 5C).

**Fig. 6:**
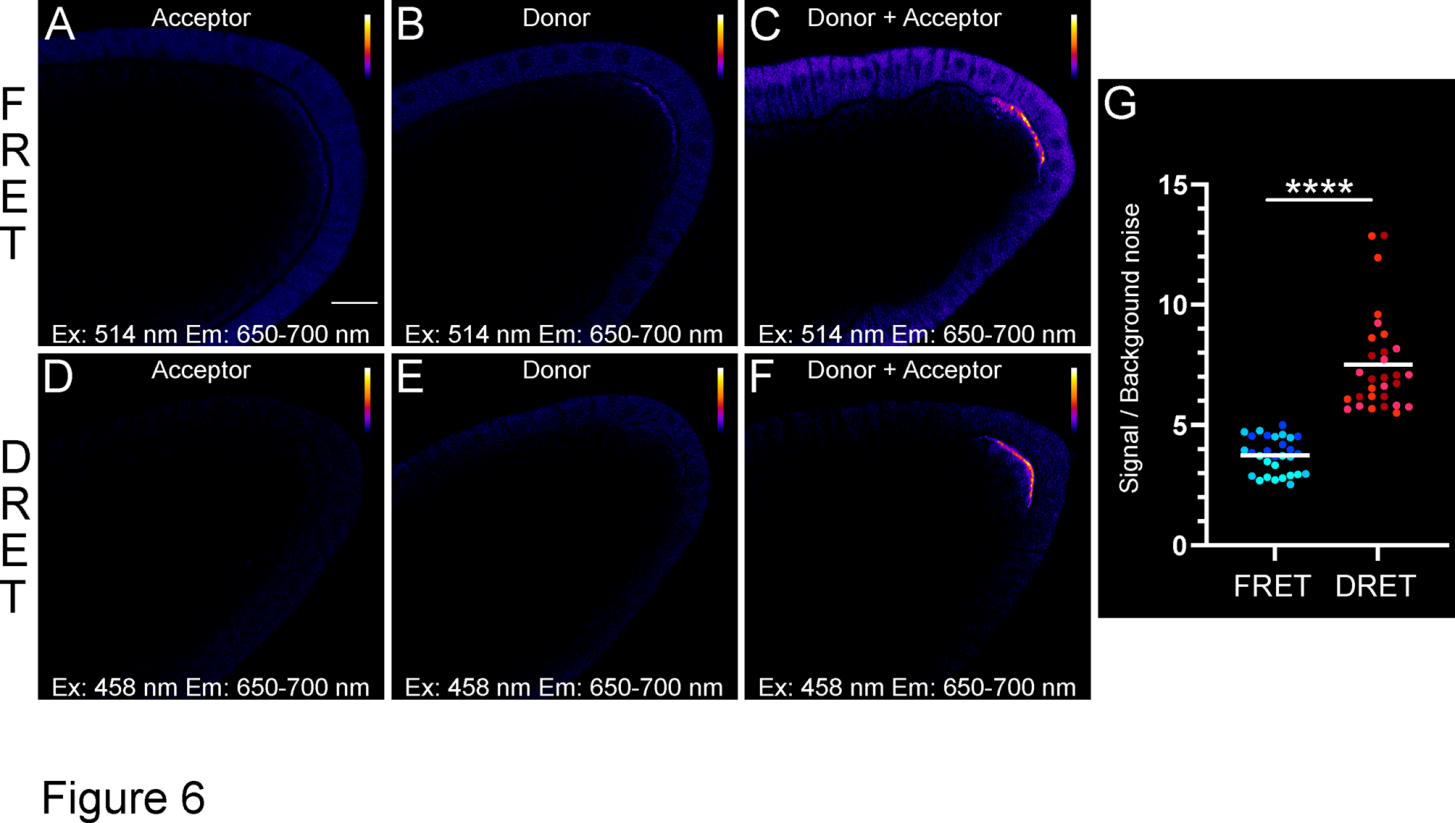
Comparative analysis of FRET vs DRET signals in stage-10A egg chambers**.** (**A**–**F**) Confocal images of stage-10A egg chambers hybridized with FRET (**A**–**C**) and DRET (**D**–**F**) pairs. FRET signals were collected after excitation at 514 nm while DRET signals were collected after excitation at 458 nm. (**A**–**C**) Signals recovered in the presence of the acceptor only **A**_**P-Cy5**_ (**A**), the donor-only **D**_**P-Cy3**_ (**B**) or the combination of both probes (**C**). A significant basal signal is observed in follicular cells and the posterior part of the oocyte. (**D**–**F**) Signals recovered in the presence of the acceptor only **A0**_**p**_ (**D**), the donor-only **D**_**p**_ (**E**) or the combination of both probes (**F**). For enhanced visualization, grey levels are color-coded using the Fire LUT. (**G**) Distributions of signal-to-background intensity ratios. Average intensities for the FRET (ex. 514 nm, em. 650–700 nm, shown in blue) and DRET signals (ex. 458 nm, em. 650–700 nm, shown in red) were normalized for each egg chambers to the average intensities of the posterior follicular cells. In total 30 egg chambers were analyzed across three distinct experiments (represented as distinct color shades) for both FRET and DRET pairs. The white horizontal lines indicate the average values of the results. Scale bar shown in (A) represents 20 µm. ****P < 0.0001 (Mann–Whitney test).

Due to the significantly higher autofluorescence at 458 nm compared to 514 nm, the auto-fluorescent signal, measured on conditions without probes, was subtracted to ensure an accurate comparison of both methods (Supplementary Fig. S12). For the FRET approach, control experiments were first conducted using the donor and acceptor probes separately. As illustrated in Fig. 6A, when excited at the donor wavelength (514 nm), the fluorescence emission from the acceptor alone is particularly strong. This observation is consistent with the notable cross-excitation of **Cy5** at this wavelength. A similar pattern, but with greater intensity, was observed when examining the donor **Cy3** on its own (Fig. 6B), characterized by a pronounced basal fluorescence emission at both the posterior pole and within the follicular cells. When the two probes were combined, a robust fluorescence signal was detected at the posterior pole of the oocyte (Fig. 6C). However, the contribution of the unhybridized probes remained significant compared to the FRET signal intensity. The SNRs, derived from 30 images (comprising 3 sets of 10 images from three distinct samples), ranged from 2.5 to 5, averaging around 3.8 (Fig. 6G).

Remarkably, shifting to the DRET approach generated significantly reduced background noise for both donor and acceptor probes when introduced separately. Excitation of the **Cy5** acceptor at 458 nm generated a fluorescence emission of very low intensity, due to the negligible cross-excitation of **Cy5** at this wavelength (Fig. 6D). A similar pattern was observed when analyzing the dark donor on its own (Fig. 6E), further validating the capacity of the intermolecular DRET strategy to almost suppress the basal signal. To then evaluate the capacity of DRET probes to detect *osk* mRNA, both donor and acceptor probes were employed simultaneously. When excited at 458 nm, a strong fluorescence signal was detected at the posterior pole of the oocyte (Fig. 6F). This sharp and well-defined signal, localized specifically at the posterior pole, contrasts with the notably weaker intensities observed in other regions of the egg chamber. As a result, the observed SNRs reached significantly higher values than for FRET probes, ranging from 5.5 to 12.8, with an average at 7.5 (Fig. 6G).

To next assess the resilience of DRET probes to irradiation, we performed photobleaching tests and compared the results with those of FRET pairs. As evidenced previously for FRET pairs^51^, photobleaching mainly depends on the photostability of the **Cy5** acceptor. Consistent with this, our analyses showed that both types of pairs displayed equivalent photostability, demonstrating their robustness and suitability for extended irradiation conditions (Supplementary Fig. S13).

To last validate the specificity of the signal generated by DRET probes, in situ* hybridization* experiments were performed using the *osk* DRET probes on egg chambers from trans-heterozygous *osk*^A87^/Df(3R)pXT103 females, in which no endogenous *osk* mRNA is produced^52^. In this context, no specific signal was detected at the posterior pole of the oocyte and only faint signal background signal was observed in the follicular cells (Supplementary Fig. S14). This underscores the specificity of the DRET probes and the unequivocal absence of hetero-DRET in the absence of the target. Collectively, these results highlight the potential of intermolecular DRET for accurate and high-contrast identification of endogenous transcripts within biological samples, and its superiority to the FRET approach.

## Conclusion

For the first time, the concept of intermolecular Dark-RET (DRET) has been applied to the *in cellulo* detection of endogenous RNA molecules. Based on Forster’s theory, we established a *R*_0_ prediction model. This model emphasizes the capacity of weakly fluorescent species to transfer energy through resonance, providing a basis to rationally design linear binary DRET probes. In light of these parameter adjustments, we integrated the water-sensitive dark donor **X** and the bright acceptor **Cy5** into 2′-OMe oligoribonucleotide sequences. From the donor and acceptor probes we tested to detect the biological *osk* RNA, a pair that triggered a 20-fold fluorescence amplification when annealed to its target was selected. We leveraged this pair to visualize *osk* mRNA within the *Drosophila* oocyte. Meeting the expectations of the background mitigation strategy, the signals from the unhybridized donor and acceptor were insignificant compared to the DRET signal operating on the target RNA. Thus, this fluorogenic method provided a clear visualization of *osk* mRNA accumulation at the posterior region of the oocyte. In contrast, the FRET approach was confronted with a significant background from the unhybridized probes, which reduced the efficiency and sensitivity of detection. Taken together, these results underscore the potential of intermolecular DRET for *in cellulo* detection of RNAs.

## Prospects

Having established the molecular bases and potential of the DRET method, it is now crucial to delve into the selection of the oligonucleotide probe backbone in order to further optimize intermolecular DRET and investigate RNAs of interest.

With the 2’-OMe-ORN probes used in this study, the conformational change from B to A influenced the distribution of surrounding water molecules around the duplex, subsequently modifying the photophysics of the donor. The challenge lies in choosing the most suitable NA backbone—one that offers robust resistance to nucleases and effectively leverages the donor’s photophysics. A plethora of candidates such as serinol derivatives, PNA, LNA, etc., provide potential options^53–58^. In addition, there is still a vast scope for enhancements in dye selection. For instance, alternative photophysical mechanisms such as TICT (twisted intramolecular charge transfer) might be the key to endow the dark donor with desired spectroscopic characteristics: remaining as quenched as possible when single-stranded and sufficiently bright once hybridized with the target to maximize energy transfer to the acceptor^59,60^. Considering the marked discrepancies in autofluorescence at 458 nm and 514 nm, designing a DRET pair with a donor that has a red-shifted absorption emerges as a strategic direction. Additionally, the chosen cyanine acceptor’s quantum yield stands at a moderate 28%^61^. Consequently, another important research avenue is to explore other acceptors devoid of cross-excitation and exhibiting higher quantum efficiencies to further amplify the DRET fluorescence signal. The resonance energy transfer capabilities of these low-fluorescence dyes hint at further application of the intermolecular DRET strategy, especially for detection of endogenous RNAs in both fixed and living cells and elucidation of their spatio-temporal regulation.

## Methods

### Probes

The target *osk* ORN, unlabeled 2′-OMe-ORNs (used as wild-type sequences), as well as Cy5-labeled 2′-OMe ORNs (employed as DRET acceptor probes) were ordered purified and ready-to-use (Microsynth AG, Balgach, Switzerland). Solid-phase 2′-OMe-ORN syntheses were performed on both an Expedite 8900 (Applied Biosystem, Waltham, MA, USA) and H-8 (K&A Labs, Schaafheim, Germany) oligonucleotide synthesizers using “trityl off” mode and ultra-mild PAc phosphoramidite chemistry on 1 μmol scale. Reagents and solvents, as well as 2′-OMe-U-CEP, 2′-OMe-Ac-C-CEP, 2′-OMe-PAc-A-CEP), 2′-OMe-iPrPAc-G-CEP phosphoramidites were purchased from Link Technologies (LGC, Teddington, Middx, UK) and ChemGenes (Wilmington, MA, USA). The standard “DMT-off” 2′-OMe RNA assembly protocol was employed except for the following modifications: 5-Ethylthio-1H-tetrazole (ETT) was employed as an activating agent; PAc-anhydride was used for capping; a longer coupling time (1200 s) was applied to the fluorenyl phosphoramidite^12^. Labeled sequences were cleaved from the solid support and deprotected with concentrated aqueous ammonia at room temperature for 12 h. Labeled 2′-OMe-ORNs were analyzed (0.5 mL/min) and purified (2.5 mL/min) by RP-HPLC—including the following apparatus: Waters™ 600 Controller with Waters™ 996 Photodiode Array Detector (Waters, Milford, MA, USA) or Jasco LC-Net II with ADC (JASCO, Tokyo, Japan)—using analytical and semi-preparative Clarity® Oligo-RP™ C18 columns with the respective dimensions: 300 × 4.60 mm and 250 × 10 mm, 5-µm particle size, 100 Å (Phenomenex, Torrance, CA, USA). The following gradient system was used: 100% A — (30 min) → 60% A/40% B — (5 min) → 100% B — (5 min) → 100% A with A = 0.9 TEAB buffer 100 mM pH 7.8: 0.1 CH_3_CN and B = 0.8 CH_3_CN: 0.2 TEAB. To prepare a 100 mM triethylamine bicarbonate (TEAB) buffer [(Et_3_NH)HCO_3_]: pass CO_2_ into a 0.1 M Et_3_N deionized aq. solution until the pH reaches about 7.8 and store at 4 °C.

### MALDI-TOF/TOF analyses of ORNs

Dibasic ammonium citrate (DAC) (98% capillary GC) and acetonitrile (HPLC grade) were purchased from Sigma-Aldrich (Saint-Louis, MO, USA). Ultrapure MALDI matrix of 3-hydroxypicolinic acid (3-HPA) was obtained from Protea Biosciences (Morgantown, WV, USA). C4 pipette tips (Zip-Tip) were from Merck Millipore (Burlington, MA, USA). Samples (500 pmol) were diluted in 10 μL of water and desalted with a C4 pipette tips (Zip-tip). The Zip-tip was activated before use with 2 × 5 μL of water:CH_3_CN (50:50) and 2 × 5 μL of DAC (50 mg/ml diluted in water). 10 μL of the ORN solution was loaded onto the Zip-tip by pulling and expelling ten times. Next, the Zip-tip was washed with 3 × 5 μL of DAC (50 mg/mL) and 3 × 5 μL of water. Elution was performed with 1.5 μL of 3-HPA matrix (80 mg/mL, 50:50 CH_3_CN:DAC) directly on the MALDI plate. The ORN profile was obtained in a MALDI-TOF/TOF mass spectrometer (AB Sciex, Framingham, MA, USA) in reflector mode with an external calibration mixture (cal mix 1 + 2 distributed by AB Sciex). MALDI-TOF/TOF–MS analysis: MS spectra were recorded manually in a mass range of 500–6000 Da resulting from 400 laser shots of constant intensity set at 6200. Data were collected using 4000 Explorer series experiments (AB Sciex).

### Optical spectroscopy characterization of labeled ORNs

#### Preparation of the samples and buffers

Labeled and non-labeled 2′-OMe ORNs as well as the target *osk* ORN were analyzed in duplicate at 2 µM in phosphate-buffered saline, pH 7.4 (50 mM sodium phosphate, 150 mM NaCl).

*Preparation of the model single-stranded solution (whole volume* = *500* *µL):* The sample was prepared by mixing 167 µL of the commercial PBS solution, pH 7.4 (*79,378 Sigma-Aldrich*), 10 µL of 100 µM **ss-ORN 1** and 323 µL of Milli-Q® water (Merck Millipore, Burlington, MA, USA).

*Preparation of the model double-stranded solution (whole volume* = *500 µL):* The sample was prepared by mixing 167 µL of the commercial PBS solution, pH 7.4 (*79,378 Sigma-Aldrich*), 10 µL of 100 µM **ss-ORN 1**, 10 µL of 100 µM **ss-ORN 2** and 313 µL of Milli-Q® water.

*Preparation of the model ternary system solution (whole volume* = *500 µL):* The sample was prepared by mixing 167 µL of the commercial PBS solution, pH 7.4 (*79,378 Sigma-Aldrich*), 10 µL of 100 µM **ss-ORN 1**, 10 µL of 100 µM **ss-ORN 2**, 10 µL of 100 µM ***ss-ORN 3*** and 303 µL of Milli-Q® water.

#### Absorption and fluorescence spectra

Absorbance spectra were recorded on a Cary 100 Bio UV–Vis spectrophotometer (Varian/Agilent, Palo Alto, CA, USA) using 500-µL cuvettes (Hellma, Müllheim, Germany) in Suprasil® quartz (Heraeus, Hanau, Germany) with 1-cm path length. Fluorescence measurements were conducted on a FluoroMax 4.0 spectrofluorometer (Jobin Yvon, Horiba, Kyoto, Japan) in a thermostatically controlled cell compartment at 20 ± 0.5 °C with slits open to 2 nm and were corrected for Raman scattering, lamp fluctuations, and instrumental wavelength-dependent bias. Emission spectra were performed with an absorbance of approximately 0.05 at the excitation wavelength corresponding to the absorption maximum of the considered fluorophore, except where specified. Quantum yields were corrected according to the variation of the refractive index of the considered medium. They were determined by comparing the integrated area of the corrected emission spectrum of the sample with that of 7-(dimethylamino)-fluorene-2-carbaldehyde in MeOH (*λ*_Ex_ = 385 nm, *Φ* = 0.46) as a reference^62^ with ± 10% mean standard deviation.

#### Denaturation studies and melting temperatures

To ensure reproducibility of hybridization and therefore measurements, the double-stranded samples were first denatured and then cooled to room temperature. Melting curves were recorded in duplicate in a Peltier-thermostatted cell holder by following the temperature-dependence of absorbance changes at 260 nm of the sample (2 μM concentration of each strand). The temperature range for the denaturation measurement was 15–80 °C. Speed of heating was 0.3 °C/min. Melting observables were converted to a plot of *α* versus temperature, where *α* represents the fraction of single strands in the duplex state. Melting temperatures (*T*_m_) were extracted from these curves after differentiation as reported elsewhere^24^.

#### Circular dichroism

Circular dichroism (CD) experiments were performed at 20 °C on a J-810 spectropolarimeter (JASCO, Tokyo, Japan). The wavelength range for CD measurements was 200–310 nm. All spectra were performed in duplicate from samples with a concentration of 2 µM for each strand. The canonical and doubly labeled ternary systems, respectively [(wt)**D**_**P**_ + ***osk.*** + (wt)**A0**_**P**_] and [**D**_**P**_ + ***osk.*** + **A0**_**P**_], demonstrated a spectral signature with two maxima: one negative at ~ 210 nm and the other positive at ~ 265 nm, typical of an A-conformation helix.

#### Calculation of transfer efficiency and signal-to-noise ratio

Transfer efficiency was determined from donor quenching in the presence of the acceptor according to Eq. S2. All measurements were performed at 2 µM. The donor-only duplex (D) denotes the donor-labeled strand hybridized to the unlabeled complementary strand; the donor + acceptor duplex (DA) denotes the donor-labeled strand hybridized to the acceptor-labeled complementary strand.

Residual donor emission spectra in the presence of the acceptor were obtained by first subtracting the acceptor-only spectrum to remove cross-excitation, followed by spectral unmixing to eliminate acceptor emission overlapping the donor fluorescence spectra.

1 $$E = 1 - \frac{{A_{D} }}{{A_{DA} }}\frac{{I_{DA} }}{{I_{D} }}$$

where $I_{DA}$ and $I_{D}$ represent the fluorescence areas of the donor in the presence and absence of the acceptor, respectively, and ${A}_{DA}$ and ${A}_{D}$ are the absorbances of the donor at the excitation wavelength in the donor + acceptor and donor only samples, respectively.

#### Fly handling

Flies were cultured under standard conditions at 25 °C. A description of the genetic markers and chromosomes can be found at Flybase. *W*1888 served as the control strain. The *oskA87* and Df(3R)pXT103 fly lines were given by Anne Ephrussi and were crossed to generate trans-heterozygous *osk* mutant females [*osk*-Gal4/UAS *osk* 3′UTR; *oskA87*/Df(3R)pXT103] which produce egg chambers in which no full length *osk* mRNA can be detected either by RT-PCR nor by classical in situ hybridization^52^.

#### In situ hybridization

Females were placed on rich food at 25 °C for at least 24 h before dissection. Fourty ovaries were dissected in ice-cold 1X PBS and then fixed for 30 min at room temperature in 1X PBS, 4% formaldehyde. After two 15-min washes with 1X PBS, ovarian chambers were dissociated by pipetting up and down and stored overnight at 4 °C in 100% ethanol. Following two 15-min washes at room temperature with wash buffer (2X SSC, 10% deionized formamide), egg chambers were incubated overnight at 37 °C in hybridization buffer (2X SSC, 10% deionized formamide, 10% dextran sulfate, 0.3 µM donor probe and/or 0.3 µM acceptor probe) and were washed twice for 30 min at room temperature with wash buffer, once for 5 min at room temperature with 2X SSC before being transferred to vectashield mounting medium and mounted between slide and coverslip.

#### Imaging

Stage-10A egg chambers were imaged with a Zeiss LSM780 NLO confocal microscope equipped with a GaAsP spectral detector, using a 40X/1.2 Apo water immersion objective. DRET signals were obtained upon excitation of the donor probe at 458 nm, collecting the acceptor emission in the 650–700 nm range. Excitation wavelength was changed to 514 nm for FRET analysis. Additionally, the acceptor probe was directly excited at 633 nm and the resulting emission was also collected in the 650–700 nm range, corresponding to the acceptor signal alone. The pixel dwell time was 6.3 µs and pixel size was 207 nm.

#### DRET and FRET signal quantification

Confocal images were analyzed using Fiji software. Three sets of experiments were conducted independently, with 10 stage-10A egg chambers analyzed per replicate. Both DRET and FRET signals were segmented by automatic Yen thresholding. The resulting masks were used to measure the mean gray values of the DRET (ex. 458 nm, em. 650–700 nm) and the FRET (ex. 514 nm, em. 650–700 nm) signals. In posterior follicular cells, mean gray values were measured within three identically-sized (40 × 40 pixels) square regions (area defined as background noise in Fig. 5B).

#### Photostability studies

The powers of the different lasers were measured at the output of the 40X 1.2W APO water immersion objective using a Thorlabs PM100A, sensor slide S170C, area of sensor 18 × 18 mm^2^. Photobleaching analysis was performed with identical laser powers, 0.35 mW on a 30.8 × 15.4 µm^2^ area (128 × 64 pixels) at the posterior pole of stage 10A egg chambers. A 6.89 zoom factor was applied on both X and Y axes. The pixel dwell time was 25.2 µs. Images were acquired every 0.794 s, over 400 s. The scan time per image was 0.206 s.

For image analysis, saturated pixels, if present, were subtracted. DRET and FRET signals were segmented through Yen thresholding. The resulting masks were used to measure the mean grey values of both DRET (ex. 458 nm, em. 650–700 nm) and FRET (ex. 514 nm, em. 650–700 nm) signals. Following normalization, the data were plotted using Fiji Curve Fitting tool to determine the parameters of the a × exp(bx) + c curve and calculate both DRET and FRET half-life values.

#### Statistical analyses

Data were plotted and statistically analyzed using Graphpad Prism 8. Groups were compared using Mann–Whitney test and statistical significance was defined as ****P < 0.0001.

## Supplementary Information

Supplementary Information.

## Data Availability

All data supporting the findings of this study are available within the paper and its Supplementary Information.
