# Supplementary material for: Intermolecular dark resonance energy transfer (DRET) for high contrast imaging of endogenous mRNAs in 3D biological samples
Source: Sci Rep. 2025 Dec 1;15:42869. doi: 10.1038/s41598-025-27000-1 (PMC12669685; doi:10.1038/s41598-025-27000-1)
Supplement: Supplementary file 1 — Supplementary Information. [file 41598_2025_27000_MOESM1_ESM.pdf]

$$k_T = \frac{1}{\tau_D}\left(\frac{R_0}{R}\right)^6 \text{ (Equation S1)}$$

Figure S1 Relationship between resonance energy transfer rate and R_0 where τ_D is the fluorescence lifetime of the donor and R the donor-acceptor distance. *When the donor-acceptor distance increases, the transfer rate constant k_T decreases sharply since it is directly correlated to this distance to the 6th power.*

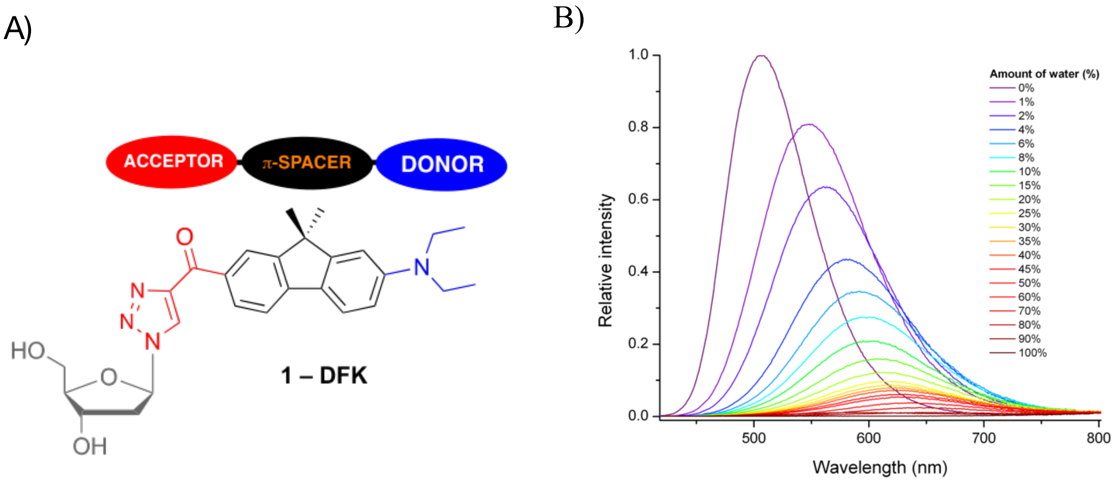

Figure S2. Fluorescence response of DFK to water. (A) Chemical structure of the push–pull nucleoside analog **DFK**—consisting of D-π-A fluorene dye as a nucleobase surrogate on a deoxyribose unit—and (B) its fluorescence emission spectra in THF upon increasing water concentration (ex. 410 nm). *Donor and acceptor functional groups are depicted in blue and red, respectively. At 10% water, only 2% of the residual fluorescence remains, demonstrating the high sensitivity of **DFK** to water.*

A

5'- GCA AAA A**X**A AAA ACG -3'
3'- CGT TTT TCT TTT TGC - **Cy5** -
5'

B

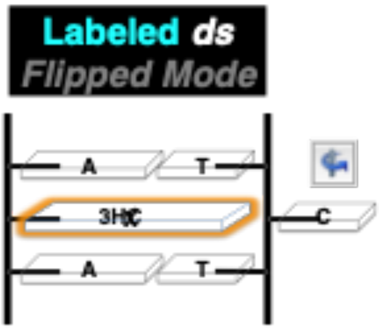

Figure S3. Structure and spatial arrangement of the modified DNA duplex. (A) Sequence composition of the [*comp-AXA* + TCT-Cy5] duplex and (B) a schematic representation depicting the spatial arrangement of the **X**-modified nucleoside intercalated into the duplex.

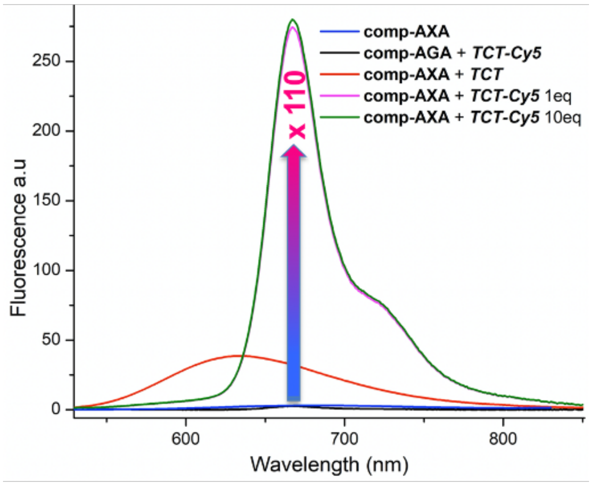

Figure S4. Energy transfer and fluorescence enhancement in labeled DNA constructs. DRET studies for ODN series: emission spectra of the [*comp-AXA* + TCT] ds-constructs in presence or absence of **Cy5** acceptor on the complementary strands. *Samples were recorded at 2 µm, in pH 7.4 PBS and excitation was performed at the donor absorption maximum. The previously studied oligonucleotide construction involved the X donor in an adenosine sandwich leading to a low quantum yield of 0.7% (blue curve, comp-AXA). After hybridization, the donor intercalates within the duplex by flipping the opposite cytidine (Figure S2). As a result, its quantum yield increases to 6.7% (red curve). In this way, the R_0 determined for this construction is 4.6 nm. Under these conditions, in the presence of the* ***Cy5*** *acceptor, resonance energy transfer is very efficient (E = 84%) and a 110-fold fluorescence enhancement is obtained (magenta curve). The presence of 10 equivalents of the acceptor probe slightly modifies the emission intensity (green curve), demonstrating the small contribution of the acceptor's cross-excitation.*

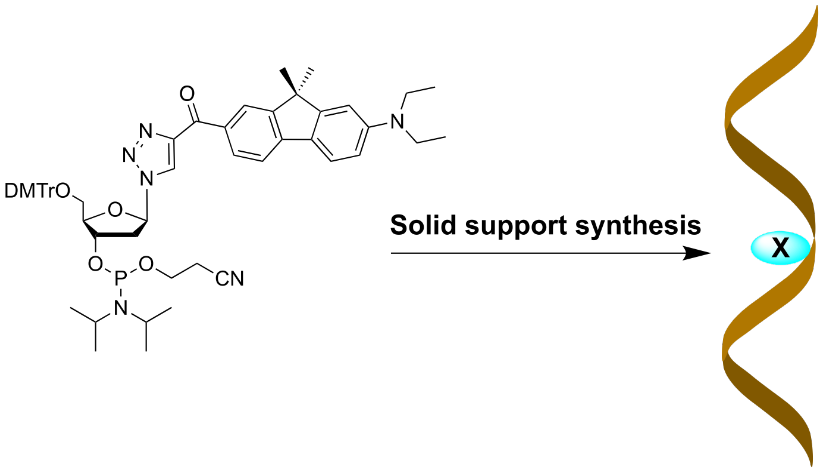

Figure S5. Chemical structure of the **X**-amidite derivative, prepared as previously reported. [1] *It was incorporated in several 2'-OMe-ORNs through solid-support synthesis.*

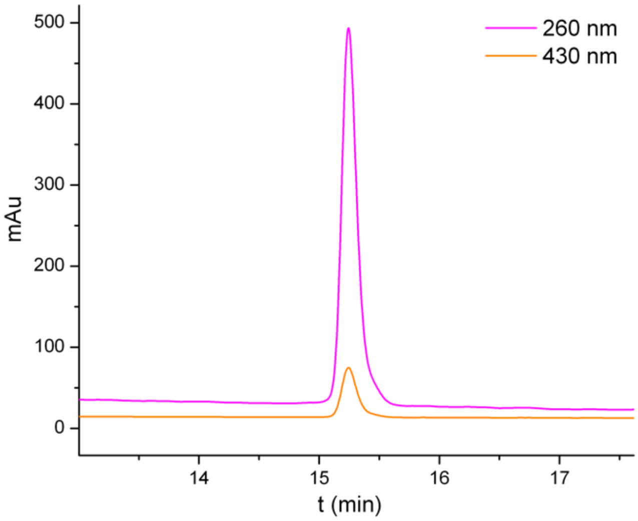

Figure S6. HPLC-based purity assessement of the donor-labeled strand. Chromatograms of the single-stranded 2'-OMe-ORN donor probe (ss-$\mathbf{D_P}$) at 260 and 420 nm.

Table S1. Sequences and analytical data of ORN probes. Target single-stranded ORN and donor-labeled single-stranded 2´-OMe-ORNs used in this study, with their respective mass and molar extinction coefficient.

ORN [a]	Sequence (5´ => 3´)	$[M+H]^+$ observed (calcd)	ε ($M^{-1}\cdot cm^{-1}$) [b]
osk.	5´-GCA CCA AUA CUU CCG CCA AAA CCUA UUA UCU UAA GUC CGU GAA AAA GC-3’	— (15247.2)	486,400
***ss*-D$_P$**	5'-GC UUU UUC ACG GAC UUA A**X**A UAA-3´	7795.1 (7796.3)	245,300
***ss*-A4$_P$**	5´-**Cy5** UUU UGG CGG AAG UAU UGG UGC -3´	7682.9 (7687.4)	221,100
***ss*-A2$_P$**	5´-**Cy5** GG UUU UGG CGG AAG UAU UGG UGC-3´	8403.7 (8405.9)	241,800
***ss*-A0$_P$**	5´-**Cy5** U AGG UUU UGG CGG AAG UAU UGG UGC-3´	9067.8 (9069.3)	264,600

a) ORNs were named according to their nature and composition depicted in Figure 4. b) Molar absorptivity of modified ORNs was calculated from www.atdbio.com/tools/oligo-calculator by considering ε_{260} = 20,000 and 12,500 $m^{-1}\cdot cm^{-1}$ for respectively **X** and **Cy5** (with **X** = **DFK** and **Cy5** = **Cyanine5**).

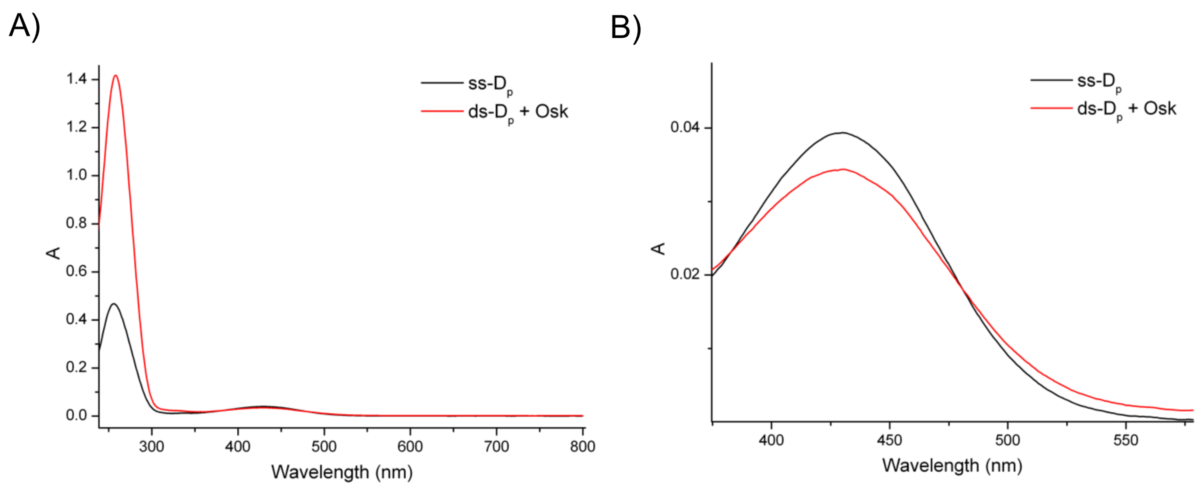

Figure S7. Absorption spectra before and after duplex annealing. (A) Absorption spectra of the single-stranded **D**$_\mathbf{P}$ and the double-stranded construct [**D**$_\mathbf{P}$ + ***osk***] in absence of the acceptor probe and (B) a zoomed-in absorption range of the **X**-modified nucleoside. *In comparison with ss-**D**$_\mathbf{P}$, the absence of λ-shift and hypochromism associated with a broadening of the width at half height demonstrate the poor intercalation of the **X** donor into the duplex.*

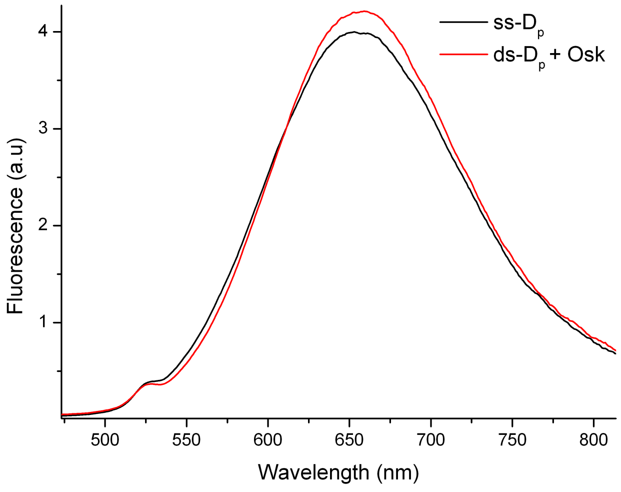

Figure S8. Fluorescence spectra before and after duplex annealing. Fluorescence emission spectra of the ss-**D**$_\mathbf{P}$ and the ds-construct [**D**$_\mathbf{P}$ + ***osk***] in absence of the acceptor probe.

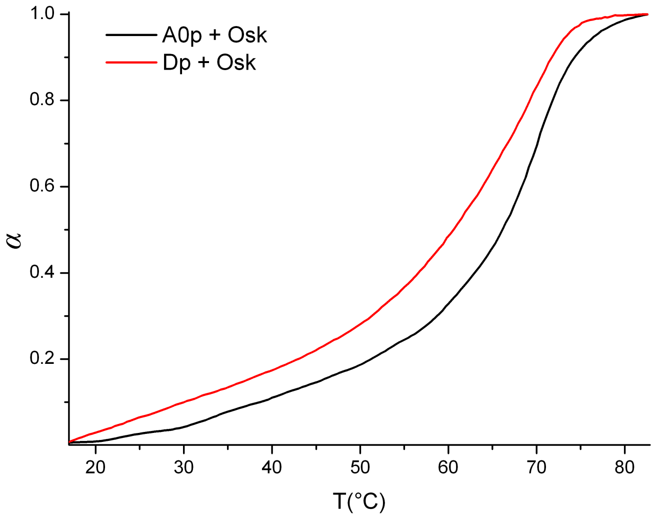

Figure S9. Duplex stability analysis. Melting temperature curves of the ds-constructs [**D**$_{\mathbf{P}}$ + ***osk***] and [**A0**$_{\mathbf{P}}$ + ***osk***].

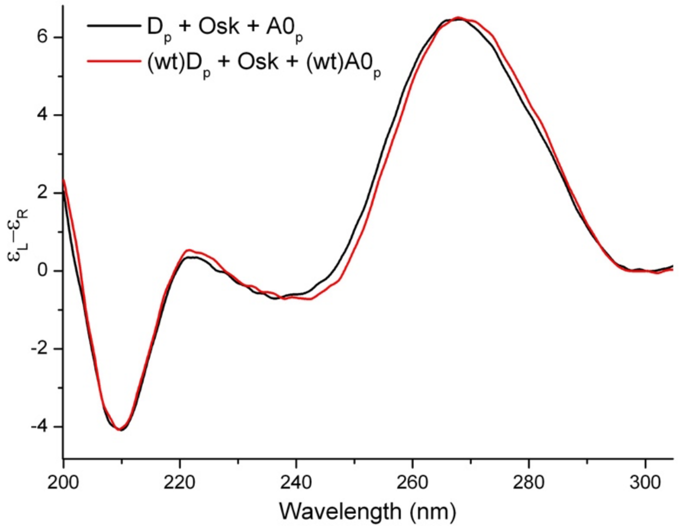

Figure S10. Conformation comparison of modified and wild-type duplexes by CD spectroscopy. CD spectra of the double-labeled ts-construct [**D$_P$** + ***osk*** + **A0$_P$**] and the corresponding wild-type [(wt)**D$_P$** + ***osk*** + (wt)**A0$_P$**].

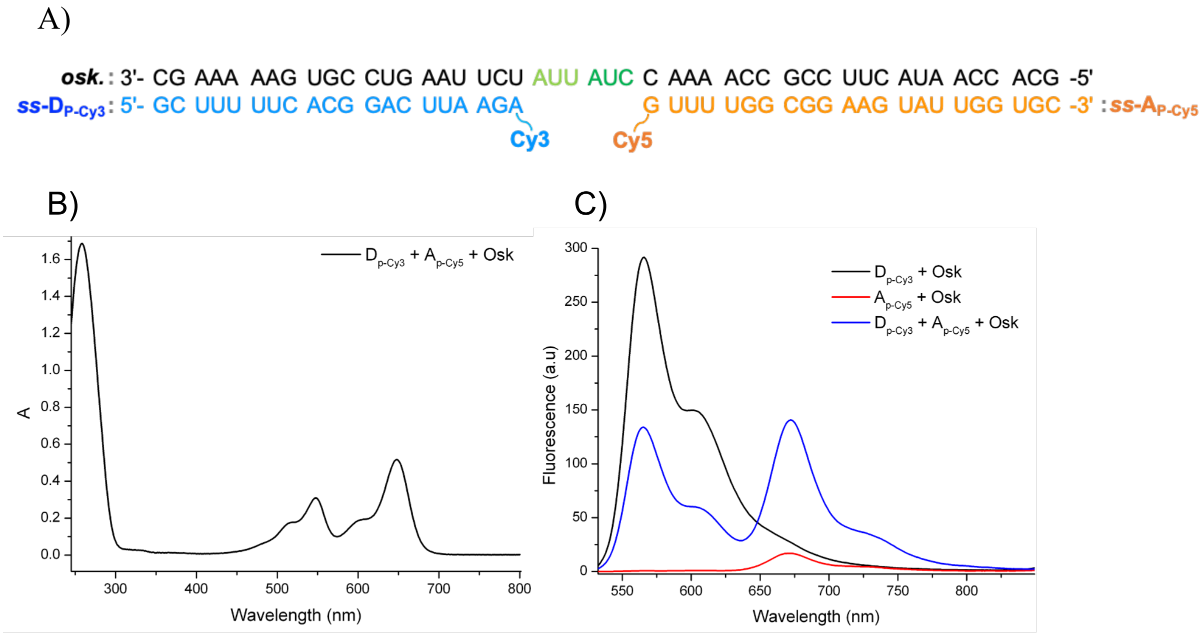

Figure S11. FRET probes photophysical characterization. (A) Graphical representation of the study system based on linear binary FRET probes for *in vitro* detection of the *osk* mRNA and (B) absorption spectrum of the ternary-stranded construct [**D$_{P\text{-}Cy3}$** + **A$_{P\text{-}Cy5}$** + ***osk***] and (C) fluorescence spectra of the double-stranded [**D$_{P\text{-}Cy3}$** + ***osk***], [**A$_{P\text{-}Cy5}$** + ***osk***] and the ternary-stranded construct [**D$_{P\text{-}Cy3}$** + **A$_{P\text{-}Cy5}$** + ***osk***] upon excitation at 514 nm, with a concentration of 2 μM for each strand. *A transfer efficiency of 55% was calculated and an amplification factor of 5 was determined at the Cy5 emission maximum.*

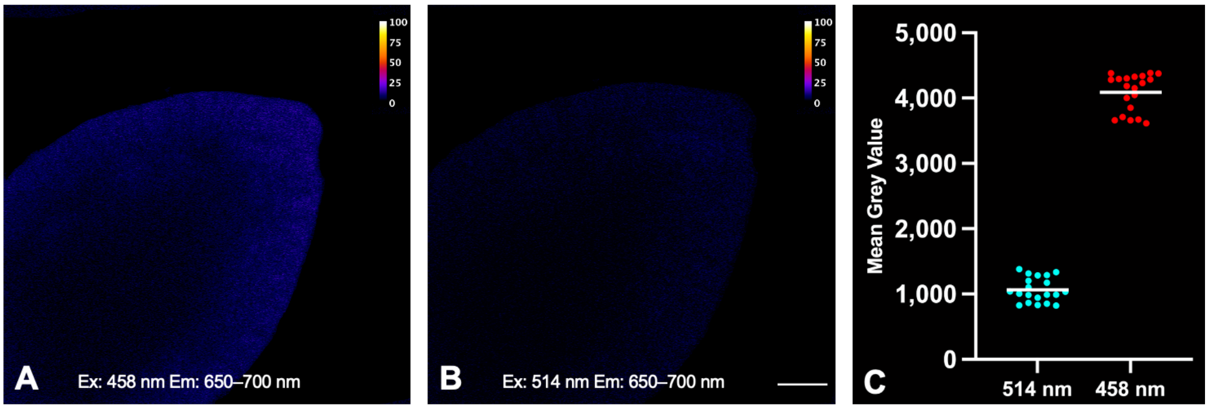

Figure S12. Comparison of background autofluorescence with 458 nm and 514 nm. Single confocal images of w[1118] *Drosophila* egg chambers without probes are shown in (A) using DRET settings (ex. 458 nm, em. 650–700 nm) and in (B) using FRET settings (ex. 514 nm, em. 650–700 nm). *The cellular autofluorescence is notably more intense at 458 nm compared to 514 nm. The average intensity within the follicular cells was measured across 20 images from four distinct analyses using the two different laser excitations.* (C) Distribution of autofluorescence intensities (shown in blue for 514 nm and in red for 458 nm). *Mean gray values were measured within three identically-sized square regions located in the posterior follicle cells. The average of the mean grey values for 514 nm and 458 nm excitation are represented by white horizontal lines. Gray levels are artificially colored using Fire Lut. Scale bar in (B) represents 20 µm.*

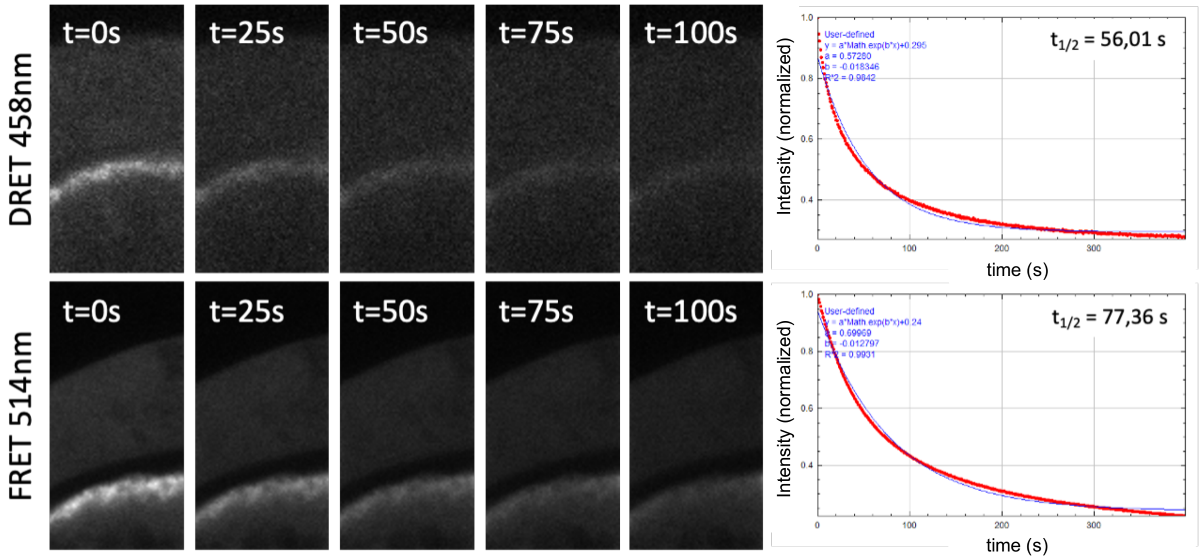

Figure S13. Photobleaching kinetics of DRET and FRET probes.
Representative images extracted at different time points of the photobleaching process. Results for the DRET pairs are presented in the top panel, and those related to FRET in the bottom one. The plots depict the mean normalized signal intensity over time, with corresponding half-life values: 56.01 s for DRET and 77.36 s for FRET*.*

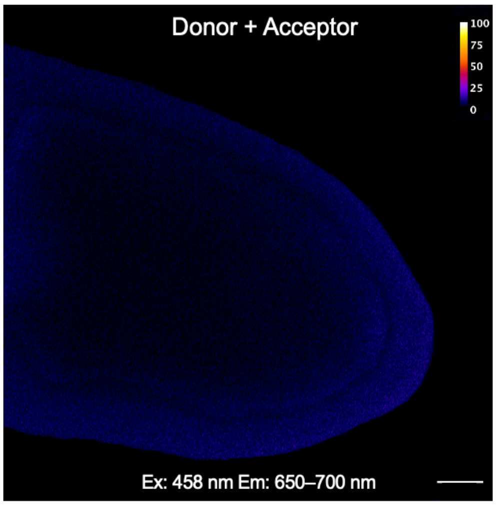

Figure S14. DRET signal obtained on *oskar*-RNA-null mutant egg chambers.
Single confocal image of a stage-10A *osk* RNA null mutant egg chamber (precise genotype: *osk*-Gal4/UAS *osk* 3’UTR; *oskA87*/Df(3R)pXT103). The signal was captured using DRET settings (ex. 458 nm, em. 650–700 nm). *The signal observed in the osk-RNA-null mutant egg chamber is comparable in intensity to that seen without probes in* w^{1118}*, indicative of autofluorescence. Scale bar represents 20 µm.*
